# CNS migration of *Diplostomum pseudospathaceum* in rainbow trout: Neuro-immunological interactions

**DOI:** 10.1007/s00436-026-08741-y

**Published:** 2026-08-05

**Authors:** Kaan Kumas, Klara Hanefeld Egeriis, Sara Kaltoft Palmelund, Magnus Marger, Kurt Buchmann

**Affiliations:** Department of Veterinary and Animal Sciences, Faculty of Health and Medical Sciences, University of Copenhagen, Frederiksberg C, Denmark

**Keywords:** Neuroimmunology, Endoparasite, Fish, Immune response, Migration

## Abstract

Rainbow trout *Oncorhynchus mykiss* serves as second intermediate host to the eyefluke *Diplostomum pseudospathaceum*, which resides as the metacercarial stage in the fish lens. The parasite uses a fish-eating bird as the definitive host and a pulmonate lymnaeid snail as first intermediate host. The fish achieves the infection when penetrated by cercariae released by the snail. The internal route of the migrating parasite (termed the diplostomule stage) in the fish (from penetration of the external surface to its destination, the eye lens) has remained partly unknown. In order to elucidate the parasite migration we exposed rainbow trout to cercarial invasion, whereafter we traced the diplostomules at selected internal locations by three approaches: (1) dissection of exposed fish and recovery of live parasites, (2) quantitative realtime PCR (qPCR) and (3) histology. We here demonstrate the presence of diplostomules in the CNS (along the spinal cord, medulla oblongata, brain) before it reaches the eye probably along the optic nerve. In the eye the parasite first enters the *corpus vitreum* and subsequently the lens. The regulation of immune relevant genes (encoding innate and adaptive immune factors) in the central immune organs (spleen and liver) and in tissues affected (CNS and eyes) by the migrating parasites were followed by qPCR. We suggest that the route taken from skin penetration via CNS to the eye elevates the probability of the parasite to survive during migration and reach its destination (eye lens) due to the low concentration of immune effector cells and molecules in CNS.

**Supplementary Information:**

The online version contains supplementary material available at https://doi.org/10.1007/s00436-026-08741-y.

## Introduction

The genus *Diplostomum* (family diplostomidae) comprises a wide array of species (Niewiadomska and Niewiadomska-Bugaj 1995; Galazzo et al. 2002; Georgieva et al. 2012; Locke et al. 2009, 2015). They all have a complex lifecycle comprising a pulmonate snail (releasing infective cercariae) as first intermediate host, fish as second intermediate host (with metacercariae in the eye lens) and a fish-eating bird as definitive host (with the adult parasite stage residing in the intestine) (Niewiadomska 1986; Chappell 1995; Capasso and Gutierrez 2023). The eyeflukes occur both in freshwater and brackish waters, where the freshwater snail is able to survive (Cichowlas 1961; Guildal 1982; Buchmann 1986).

Several species are known to affect both wild and cultured fish populations, because the presence of metacercariae in the fish may cause disease (Shariff et al. 1980; Chappell 1995; Lyholt and Buchmann 1996; Buchmann and Bresciani 1997; Karvonen et al. 2004, 2006; Duan et al. 2021). Following penetration of the fish host surface and shedding of the tail the cercaria attains the diplostomule stage and initiates a fast migration towards the eye. The diplostomule reaches and attains the metacercarial stage in the lens, which is believed to be an immune-priviledged site (Höglund and Thuvander 1990; Fuad et al. 2024). If the cercarial exposure is sufficiently high in relation to the fish size the cercarial penetration of fish surfaces may elicit acute mortality (Larsen et al. 2005). Chronic infections elicit cataract (Shariff et al. 1980), decrease growth rate due to impaired feed intake (Buchmann and Uldal 1994) and elevate the risk of predation (Seppälä et al. 2004, 2006).

The migration route taken by the diplostomules, from the cercariae enter the host through epidermal surfaces until they reach the lens, is still uncertain. Fish exposed to isotope tagged cercariae of *D. spathaceum* indicated that gill surfaces and to some extent skin and fins were the main penetration sites (Erasmus 1959; Ratanarat-Brockman 1974; Höglund 1991) and it has been hypothesized that the parasite reaches the eye lens via blood vessels (Ferguson 1943; Betterton 1974) or even migrate through muscles and connective tissues (Hoffmann and Hoyme 1958; Erasmus 1959). Proteinase production in *Diplostomum* cercariae may assist movements in dense tissues (Moczon 1994), but the short time from penetration to lens indicates that easier ways can be chosen. We have elucidated the migration route in further depth.

We established an infection model consisting of rainbow trout *Oncorhynchus mykiss* serving as second intermediate host, and as parasite we applied cercariae of *Diplostomum pseudospathaceum* released from the pulmonate snail *Lymnaea stagnalis.* The eyefluke *D. pseudospathaceum* is one of the most common parasites in fish and snails from Danish freshwater lakes (Haarder et al. 2013; Duan et al. 2021), and it is therefore relevant to study the impact of this parasite on fish.

In order to follow the migration route of the parasite after penetrating the fish surface we applied classical fish parasitological methods (dissection of fish and recovery of live parasites), histology and quantitative realtime PCR (qPCR). We could follow the diplostomules, and we here describe the infection route from the penetration site (skin) via the central nervous system (CNS) to the eye compartments. We suggest that a migration route along and through a relatively immune-priviledged system, such as CNS, and along the optic nerve to the eye entrance will optimize the speed and success of the migration of the diplostomule.

We hypothesize that the CNS and interior eye compartments act as immune-priviledged organs for the parasites, but we suspect that the fish immune system, during the early migration phase of the parasite, may be affected to some degree as previously indicated (Höglund and Thuvander 1990; Fuad et al. 2024). During a 7 h infection process we therefore investigated regulation of a series of genes (by use of quantitative realtime PCR) known to be involved in rainbow trout immune reactions against bacterial and parasitic pathogens (Kania and Buchmann 2024).

## Materials and methods

### Ethics and license

The work was conducted under the Animal Experiments Inspectorate license 2024-15-0201-01697 (Ministry of Food, Agriculture and Fisheries). Care was taken to reduce pain and suffering of animals. They were continuously monitored, and if clinical disease signs were observed, the animals were euthanized immediately.

### Fish tanks and water source

Fish were kept in fish tanks with a total volume of 120 L in constant artificial light. The water temperature was kept constant at 19℃ by performing the trial in a thermostat controlled experimental room. Water used was constantly aerated municipal water (near 100% oxygen saturation) from Frederiksberg Commune and recirculated with internal filters (Eheim, Germany). The Frederiksberg municipal water had a pH 7.6, a calcium carbonate content of 490 mg/L fish. Ammonia and nitrite levels were measured by dip sticks (Merck, Germany) and kept below 1 mg/L and 0.5 mg/L, respectively.

### Parasites

**Fig. 1:**
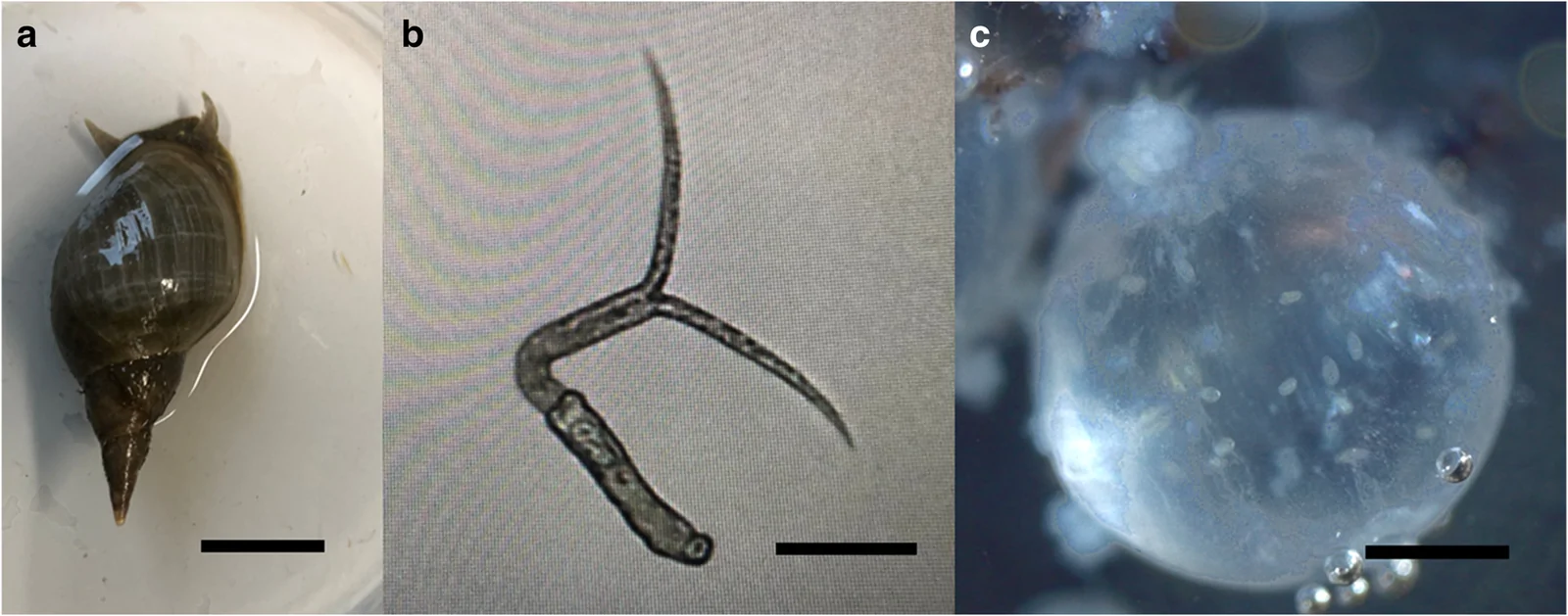
**a**. Intermediate host *Lymnaea stagnalis* used for harvest of cercariae. Scale bar: 1 cm. **b**. *Diplostomum pseudospathaceum* cercaria shed from the snail. Scale bar: 300 μm. **c**. *d. pseudospathaceum metacercariae* in lens of rainbow trout 7 hpi. Scale bar: 900 μm.

A pulmonate snail, *Lymnaea stagnalis*, (Fig. 1a) was collected in the freshwater lake Søndersø (Værløse, Zealand, Denmark), September 2025 and was placed in a 100 mL beaker with 20 mL filtered (0.45 μm) fish tank water at room temperature. The snail was infected, whereby cercariae (Fig. 1b) were readily shed into the water. Water was replaced several times over the day allowing cercariae (thousands released per hour) to be collected by pipetting under the dissection microscope (Leica, Germany) and used for the infection process. This started with cercarial penetration of fish surfaces followed by internal diplostomule migration resulting in lens residing metacercaria (Fig. 1c). Subsamples were conserved in 96% ethanol for subsequent molecular identification.

### Parasite species identification

Cercariae were used for the identification procedure. This was based on a subsample of conserved parasite larvae, which were subjected to DNA purification, PCR and sequencing of rDNA, the internal transcribed spacer (ITS) (Haarder et al. 2013; Duan et al. 2021). In brief: DNA extraction was achieved by using QIAamp DNA Mini Kit (cat.no. 61306, Qiagen, Hvidovre, Denmark. PCR was performed using BioRad T100 Thermal Cycler (cat.no. 1861096, Bio-Rad Laboratories, Denmark). We applied a 60 µL reaction volume containing 6 µL of 100 mM dNTP mix (Applied Biosystems™ GeneAmp™ dNTP Blend, cat.no. 10085714, Fisher Scientific, Slangerup, Denmark), 28.6 µL of DNase and RNase-free water (cat.no. 12060346, Fisher Scientific, Denmark), 6 µL (each) of 10 mM forward and reverse primers (TAG Copenhagen, Frederiksberg, Denmark), 1.8 µL of 50 mM MgCl2, 0.6 µL of DNA Polymerase, 6 µL of 10× Reaction buffer (BIOTAQ DNA Polymerase, cat.no BIO-21060, Saveen & Werner ApS, Jyllinge, Denmark), and 5 µL of parasite DNA. The internal transcribed spacer (ITS) region was targeted by forward primer PDG_18S_F5 (5′-GATAACGAACGAGACTC-3′) (Wan Sajiri et al. 2024) and reverse primer NC2 (5′-TTAGTTTCTTTTCCTCCGCT-3′) (Gasser et al. 1993). The reaction was based on pre-denaturation at 94 °C (5 min), 40 cycles of denaturation (95 °C) for 30 s, annealing (54 °C) for 30 s, elongation (72 °C) for 2 min and post-elongation (72 °C) for 7 min. PCR products were run in a 2% agarose gel and purified by using Illustra™ GFX™PCR DNA and Gel Band Purification Kit (cat.no. 28-9034-71, VWR International A/S, Søborg, Denmark). Sequencing was performed by Macrogen Europe, the Netherlands, and sequences were analyzed by CLC-Main Workbench v20.0.0.4 (QIAGEN, Hvidovre, Denmark). Sequences were submitted to GenBank and assigned accession number PX675814.

### Fish

**Fig. 2:**
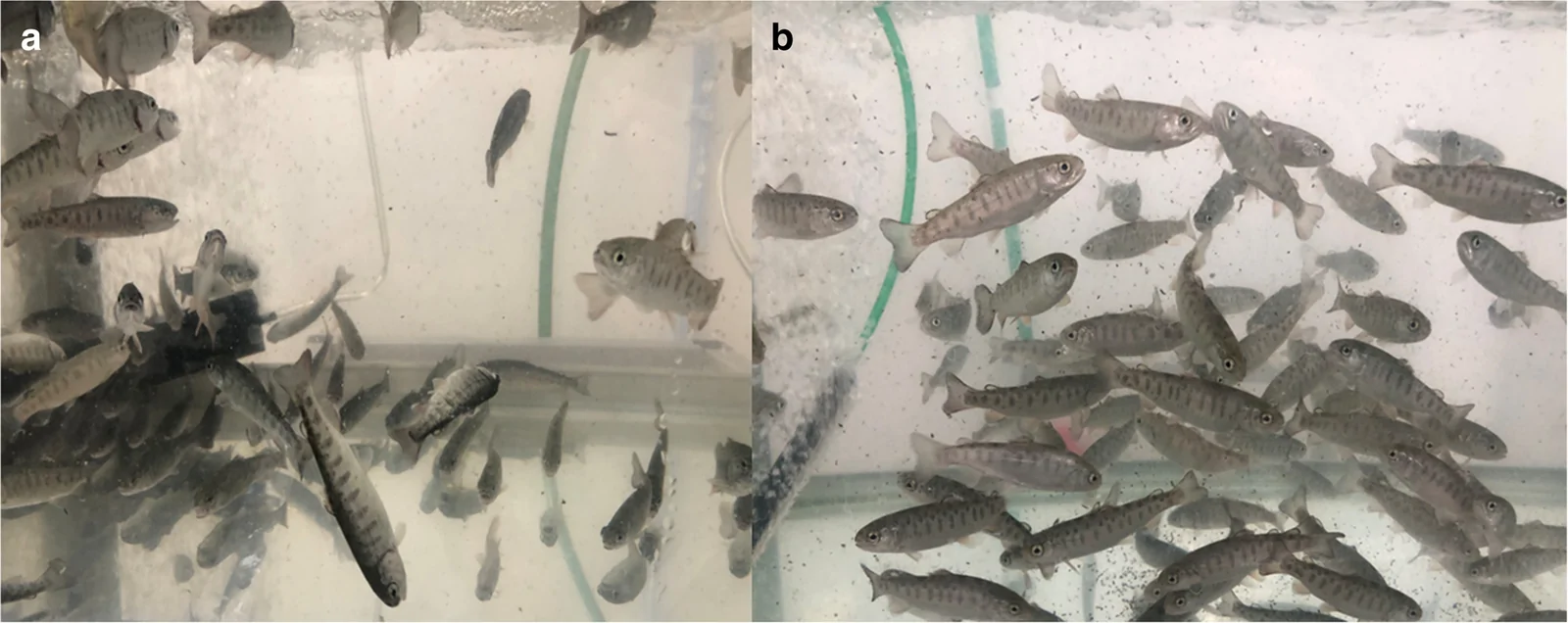
Rainbow trout juveniles used for experimental exposure to *Diplostomum pseudospathaceum* cercariae. **a**. Equilibrium disturbances of rainbow trout following cercarial exposure (600 parasites per fish) 7 h post-infection (hpi), **b**. Non-exposed control rainbow trout at same time point

We used an outbred strain of rainbow trout *Oncorhynchus mykiss* supplied by the company Aquasearch ova ApS (Jutland, Denmark). Certified and disease-free eggs were disinfected, incubated, hatched and fish subsequently reared at the Bornholm Salmon Hatchery (Nexø, Bornholm, Denmark), which is a recirculated disease-free system (Xueqin et al. 2012). When reaching a size of 5–6 g juveniles were transported to the infection facility at Frederiksberg Campus (Capital region of Denmark). Before experimentation was initiated, we secured a two-week acclimatization period, with feeding (1% of biomass per day) using commercial pelleted feed (Inicio, Biomar, Denmark). The infection trial lasted 7 h (Fig. 2a, b).

### Exposure of fish to parasites

Experimental rainbow trout were exposed together in one fish tank with added newly released cercariae. A total of 76 fish were exposed to cercarial infection at 19 °C in a 120 L glass aquarium with a content of 100 L constantly aerated water with cercariae (600 per fish), which had been collected and concentrated in a volume of 500 mL water. A similar aquarium with 76 fish was kept as non-infected control (sham infected by adding 500 mL parasite free mL water). Time points for sampling of exposed fish (10 fish per sample) and non-infected time point control fish (10 fish per sample from the non-infected tank) were 1, 2, 3, 4, 5, 6 and 7 h post-infection (hpi).

### Euthanasia of fish

Fish were euthanized by an over-dosage of anaesthetics (immersion into a solution of 300 mg/L tricaine-methane sulphonate MS222, Sigma-Aldrich) and immediately subjected to dissection and sampling of organs (Buchmann 2007).

### Organ sampling

The sampling time protocol is depicted in Table 1. Euthanized fish were placed on the dissection table and opened (ventral and lateral incisions exposing the body cavity with organs) by use of scalpel, scissors, and forceps. For the gene expression study, we sampled at time points 1, 2, 4, 6 and 7 hpi the spleen and liver (from ten fish), eyes (one eye from each of five fish) and brain (from the same five fish). Samples were transferred to RNAlater for subsequent RNA purification/ gene-expression studies. For the molecu­lar detection of parasites, we sampled the same number of eyes (5) and brains (5) for DNA extraction and PCR. For the histology study, we recovered at each time point one eye and one brain from each of two fish. We fixed them in neutral formalin for subsequent paraffin embedding. The brain was recovered by sectioning the upper dorsal epaxial part of the head from snout to neck. Hereby the brain was kept intact in the cranium with external covering of skin and musculature. For the live parasite monitoring study, we removed the eyes (by cutting first the epidermis around the eye and subsequently the optic nerve) from three fish at each time point and placed them in separate Petridishes with 2 mL water. Eyes were sectioned with a scalpel allowing separation of corpus vitreum and lens, whereafter diplostomules were counted under the dissection microscope. For the live para­sites in brain study, we sampled six fish (only at 7hpi). The cranial roof was cut and lifted by a scalpel, whereafter the brain was removed by forceps and examined in fresh and un-conserved form under the dissection microscope for enumeration of live parasites (diplostomules).

**Table 1:** Sampling protocol. Number of fish sampled at each time point for specific purposes. Codes: hpi: hours post exposure, DP-DNA: *Diplostomum pseudospathaceum* DNA, LM: light microscopy

Time points	Live parasite monitoring in eyes	Live parasite monitoring in brain	Spleen for gene expression	Liver for gene expression	Eyes for gene expression	Brain for gene expression	Eyes for DP-DNA	Brain for DP- DNA	Eyes for histo-logy	Brain histo-logy
1 hpi	3(6 eyes)	-	10	10	5	5	5	3	2	2
2 hpi	3(6 eyes)	-	10	10	5	5	5	3	2	2
3 hpi	3(6 eyes)	-	-	-	-	-	5	3	2	2
4 hpi	3(6 eyes)	-	10	10	5	5	5	3	2	2
5 hpi	3(6 eyes)	-	-	-	-	-	5	3	2	2
6 hpi	3(6 eyes)	-	10	10	5	5	5	3	2	2
7 hpi	3(6 eyes)	6	10	10	5	5	5	3	2	2
Fixation/ Method	Fresh LM	Fresh LM	RNAlater/ qPCR	RNAlater/ qPCR	RNAlater/ qPCR	RNAlater/ qPCR	RNA later /qPCR	RNA Later /qPCR	Formalin/ Paraffin	Formalin/ Paraffin

### Detection of parasites by histology

Material fixed in neutral formalin was processed through graded ethanol series, xylene substitute and embedded in paraffin before sectioning. Section  (4 μm) were mounted on microscope slides and stained by haematoxylin and eosin, dehydrated and mounted in Depex. Slides were studied in a light microscope (Leica DM, Germany) and photos captured by Leica LAS.

### Detection of *Diplostomum pseudospathaceum* DNA in brain and eye tissues by qPCR

In order to trace diplostomules following cercarial exposure we isolated DNA from brain and eyes of rainbow trout sampled during the exposure trial. *Corpus vitreum* and eye lenses were analyzed separately.

#### DNA extraction

DNA from fish tissues (brain, eye) were extracted by using QIAamp DNA mini kit (Qiagen, Denmark) according to the manufacturer’s instructions with the modification of using 50 µL AE buffer instead of 200 µL. For each eye sample the *corpus vitreum* was lysed first, whereas the more hard lens was mechanically disintegrated before lysis.

#### RT-PCR analysis

*D. pseudospathaceum* DNA was detected by amplifying the internal transcribed spacer (ITS) region of ribosomal DNA (rDNA) using forward primer NC5 (5′-GTAGGTGAACCTGCGGAAGGATCATT-3′), reverse primer DP-ITS1-R7 (5′-ACGCAGCAGAACTACGTCTC**-**3′) (this study) and probe DP_ITS1 (5′-GCGTGTCAAGGAATTGACGGATGGG**-**3′) (designed for this study). The RT-PCR analysis was performed using AriaMx Real-Time PCR Systems (Agilent). Each reaction consisted of 2.75 µl DNase/RNase free H_2_O, 6.25 µl Brilliant III Ultra-Fast QPCR Master Mix (AH Diagnostics AS, Denmark), 1 µl primer-probe mixture (primer: 10 µM, probe: 5 µM) and 2.5 µl sample DNA. The reaction’s thermal profile was 1 cycle of pre-denaturation step for 3 min at 95 °C, followed by 40 amplification cycles consisting of 5 s denaturation at 95 °C followed by 60 s elongation at 60 °C. These qPCR assays were run in duplicate and only samples giving amplification in both plates were taken into consideration.

#### Determination of qPCR detection sensitivity and efficiency of the DNA detection method

In order to assess the sensitivity of the qPCR assay, a ten-fold serial dilution series was prepared from a 10 ng/µL DNA extracted sample of *D. pseudospathaceum*. The dilution series ranged from 10⁰ to 10⁻⁸, and a total of eight independent replicates were tested for each dilution. Additionally, we also tested fish DNA and No Template Control (NTC), which contained no DNA) (Supplementary file S2.1). qPCR assays run as described above.

#### Expression of immune relevant genes

We followed the activation of selected immune relevant genes in brain and eyes by qPCR. The list of genes studied and the primers and probes used in the process are listed in supplementary Table S1. In brief: we targeted genes encoding interleukins (IL-1β, IL-2, IL-4/13α, IL-6, IL-8, IL-10, IL-12, IL-17 A/F2, IL-17C1, IL-17C2, IL-22), type II interferon (IFNγ), tumor necrosis factor α (TNFα), serum amyloid A (SAA), complement factor 3 (C3), immunoglobulins (IgM, IgDm, IgT, IgDs), cathelicidin (Cath-1), lysozyme, and cellular markers including T cell receptor β (TCRβ), major histocompatibility complex molecules (MHC I, MHC II), T cell markers (CD4, CD8). As reference genes we measured the ARP, B-actin, and ELF-1α by using the average of the Cq values based on NormFinder. All qPCR assays were performed in the AriaMx™ real-time PCR system (AH diagnostics AS, Denmark) using the following conditions: 95 °C for 3 min, 40 cycles of 95 °C for 5 s and 60 °C for 15 s, followed by endpoint measurement. The qPCR results were analyzed using the simplified 2-ΔΔCq method as all the qPCR assays had efficiencies within ± 5%.

### Statistics

GraphPad Prism version 11.0.0. (San Diego USA) was used data analysis. Assessment of normality and log-normality we conducted the Kolmogorov-Smirnov test. In cases that followed a normal distribution, one-way ANOVA and two-way ANOVA with Šídák’s multiple comparisons tests were applied. We found that efficiencies of 100 ± 5% was measured for all q-PCR assays, which allowed us to use the simplified 2^−∆∆^ Cq data analysis (Livak and Schmittgen 2001). For normalization and as internal calibrator we used the average of two (best performing) of the three genes (ARP, ELF1a, and b-actin) (NormFinder by Andersen et al. 2004). For the qPCR data Student’s t-test was used to compare exposed and non-exposed fish. Differences were found significant when regulations were at least ± 2 two-fold and a two tailed student T-test with Welch’s test comparisons showed *p* < 0.05.

## Results

### Molecular identification of the parasite

The parasites (cercariae) used for infection of rainbow trout were subjected to molecular identification (PCR and sequencing), whereby it was established that the eye fluke species was *Diplostomum pseudospathaceum.* PCR amplified the rDNA (ITS) region and gained a product of 1688 bp (including primer binding sites) and gained GenBank accession number PX675814. Blast analysis at NCBI confirmed identity with the closest identities of other isolates of the species ranging from 99.92% to 99.76% (GenBank accession numbers OK329927.1 and MW000985.1, respectively).

### Detection of live parasites in trout

*Eyes:* Live diplostomules/metacercariae were detected by light microscopy in the eyes of rainbow trout already within 2 hpi, where two out of three fish were found infected with metacercariae in the lens (Fig. 1c). Already at 4 hpi all of three examined fish were infected and the number of metacercariae increased steeply until 7 hpi (110 metacercariae in three fish), where the trial was terminated (Tables 1 and 2). The exposure experiment was terminated at 7 hpi due to detection of clinical signs, especially equilibrium disturbances (whirling) (Fig. 2a), whereas non-exposed fish showed no adverse reaction (Fig. 2b).

**Table 2:** Live metacercariae (no. in lens/no. in vitreous humor) at each time-point in eyes of rainbow trout exposed to *Diplostomum pseudospathaceum* cercariae. Three fish examined per time point (hours post-infection: hpi)

	Fish 1		Fish 2		Fish 3		
Time point	Left eye	Right eye	Left eye	Right eye	Left eye	Right eye	Total
0 hpi	0	0	0	0	0	0	0
1 hpi	0	0	0	0	0	0	0
2 hpi	0	2/0	0	0	1/0	0/1	4
3 hpi	0	0	0	0	1/0	1/0	2
4 hpi	1/1	2/0	2/2	2/1	3/2	1/0	17
5 hpi	4/0	4/3	7/6	4/3	3/0	5/2	41
6 hpi	4/6	5/7	12/7	10/6	14/6	16/5	98
7 hpi	16/9	12/9	14/1	6/10	11/4	12/6	110

*Brain:* At 7 hpi a total of six fish, which had been exposed to parasites, were euthanized. The cranial roof was cut off with a scalpel, the brain removed and examined in a Petri dish with water under the dissection microscope. Haemorrhages were evident. In two of the six brains live diplostomules were detected (4 and 6 diplostomules/metacercariae, respectively). Non-infected control fish did not present with similar haemorrhages.

### Histological detection of diplostomules/metacercariae in rainbow trout

**Fig. 3:**
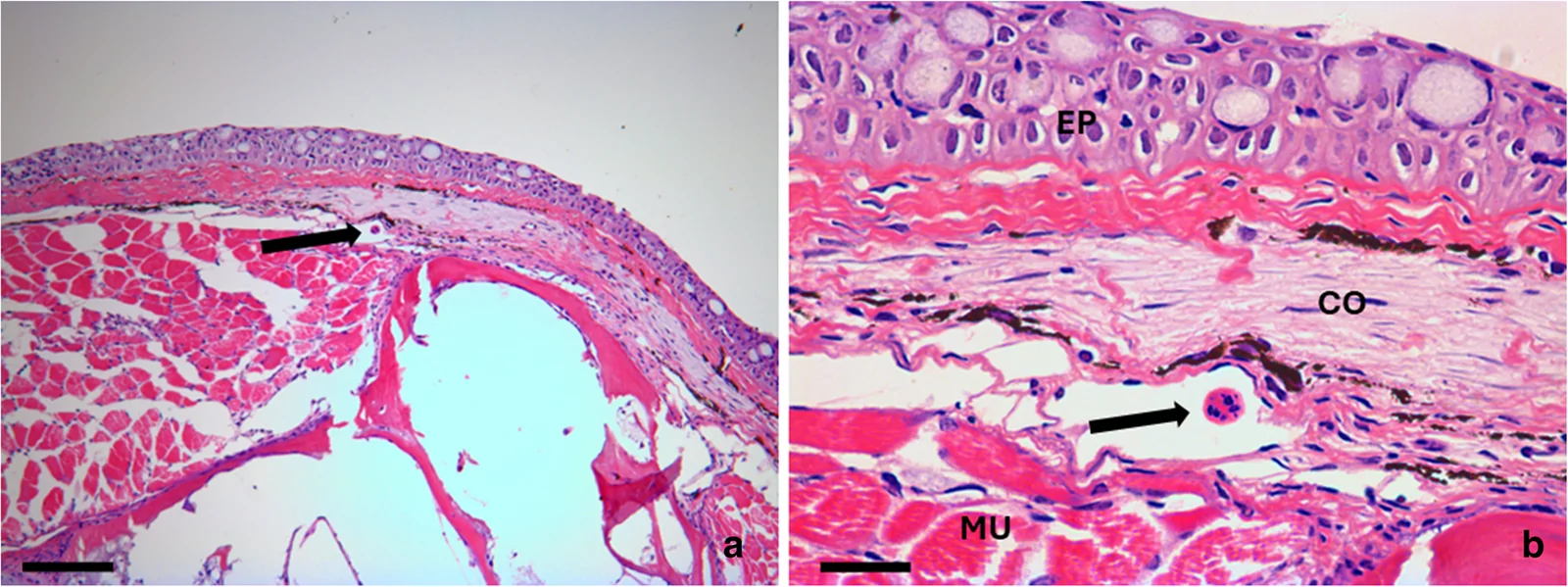
*Diplostomum pseudospathaceum* diplostomule (arrow) in skin (head of rainbow trout. **a**. Subcutaneous location. Scale bar:100 μm. **b**. Higher magnification of the same parasite located subcutaneously. Scale bar: 25 μm. MC: Mucous cells, EP: epidermis, CT: Connective tissue, MU: Muscle tissue

*Parasites in subcutis*: Haematoxylin stained histological sections (total 288 sections) of rainbow trout (dorsal part of head examined) exposed to cercariae were investigated at 2–7 hpi. In these Sect.  4 diplostomules were detected at subepidermal and 6 at other subcutaneous locations (Fig. 3a, b).

**Fig. 4:**
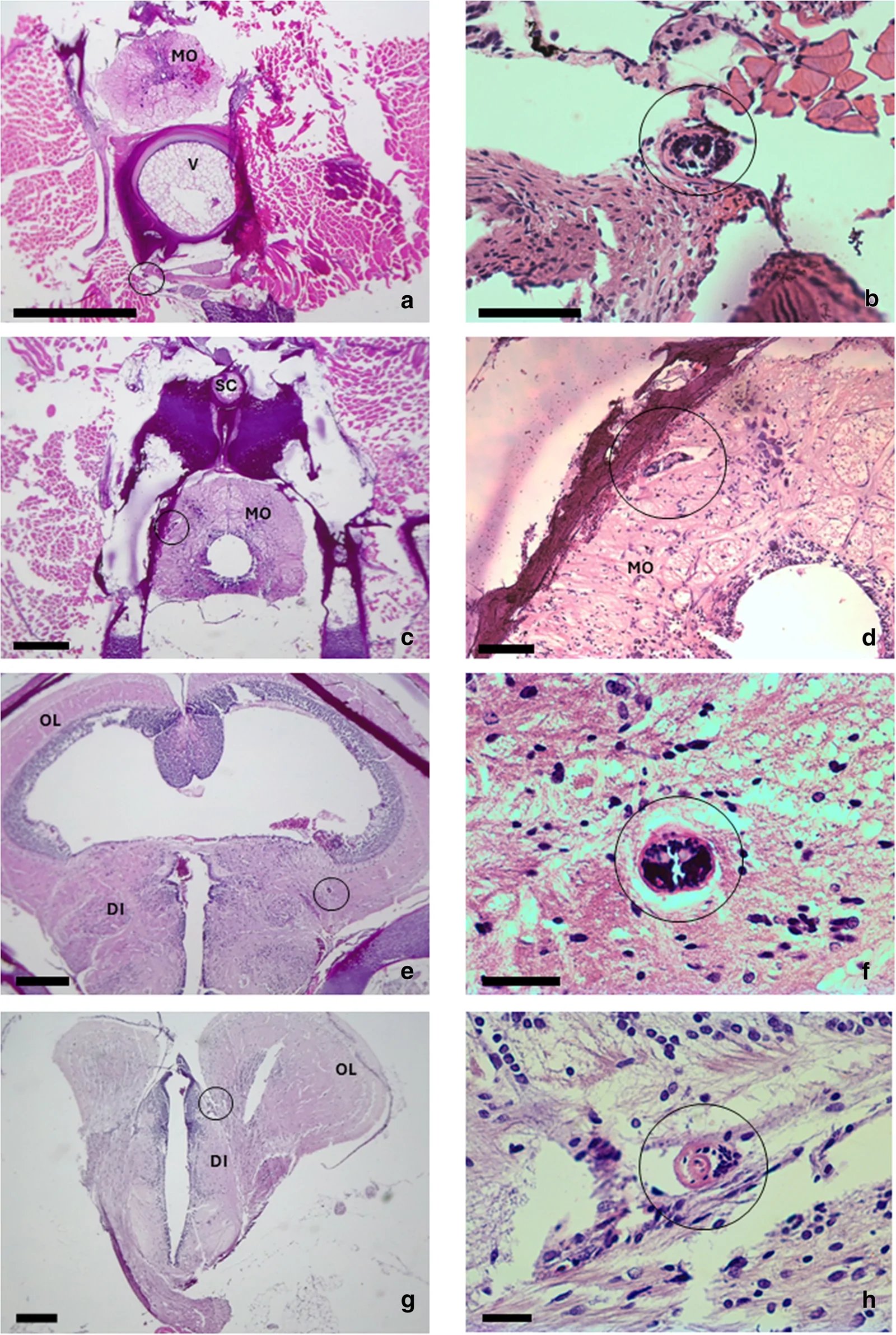
Diplostomules of *D. pseudospathaceum* (encircled) in CNS structures. Sections are taken in a caudal to cranial direction (**a**–**h**). (**a**) Occurrence of the parasite near the spinal column. V: Vertebra (cross-section). M: Medulla. Scale bar: 800 μm. (**b**) Higher magnification of the same parasite. Scale bar: 50 μm. (**c**) Parasite located inside the *medulla oblongata* (MO). SC: Spinal cord location. Scale bar: 400 μm. (**d**) Higher magnification of same C section. Scale bar: 110 μm. (**e**) Parasite located in the Diencephalon (DI) and optic lobes (OL). Scale bar: 380 μm. (**f**) Higher magnification of the same E section. Scale bar: 37 μm. (**g**) Occurrence of the parasite inside DI. Scale bar: 300 μm. (**h**) Higher magnification of the same G section. Scale bar: 25 μm

*Parasites in CNS:* Deeper CNS locations were along the *medulla*,* medulla oblongata* (Fig. 4a–d) and brain under the cranium (Fig. 4e–h). Penetration of diplostomules into brain tissue was observed (Fig. 4e–h). A total of 288 sections of fish heads (comprising skin, subcutis and brain) were examined for presence of diplostomules. The number of sections with parasites detected by histology of CNS tissues was 49. However, some of these were neighbouring sections of the same parasite. Care was therefore taken to record only individual full parasites (Table 3).

**Fig. 5:**
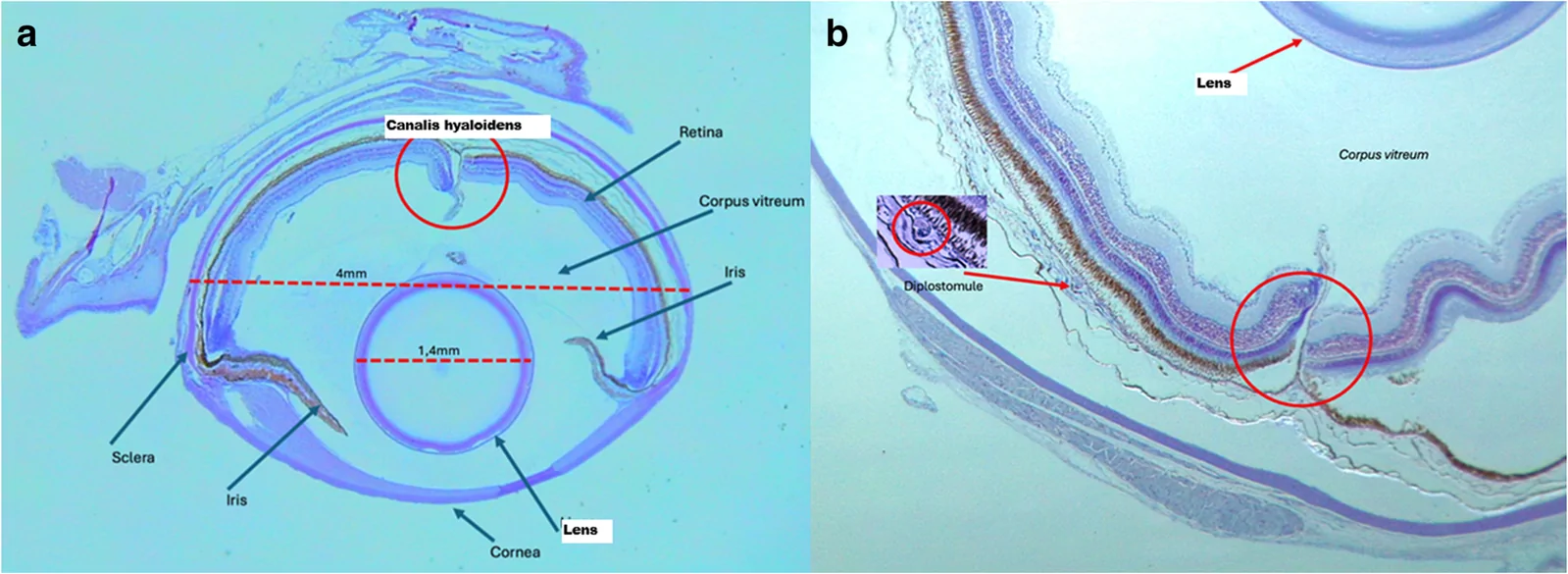
**a**. Rainbow trout eye section with dimensions (overview) showing anatomical structures. **b**. Diplostomules located near entrance site along *canalis hyaloides*

**Fig. 6:**
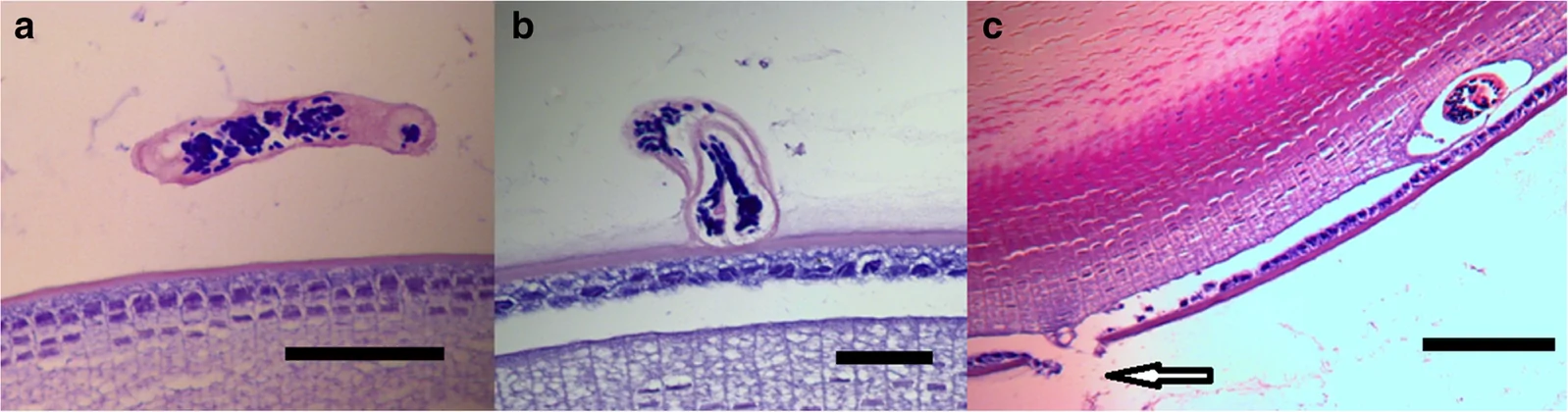
Diplostomules/metacercariae located in eye of rainbow trout. **a**. Location in *corpus vitreum* after entry. Scale bar: 50 μm. **b**. Attaching diplostomule to external face of lens capsule. Scale bar: 30 μm. **c**. Diplostomule inside lens following lens capsule penetration. Scale bar: 60 μm

*Parasites in eyes:* A total of 455 sections of eyes were examined (Table 3). These were distributed as 65 sections of eyes taken at each time point (1–7 hpi). No parasites were detected at 1 and 2 hpi but from 3 hpi they occurred frequently. When reaching the eyes diplostomules were found at the back of the eye (Fig. 5a), at the entrance site of the optic nerve, next to the *canalis hyaloidens* (Fig. 5b), in the *corpus vitreum* (Fig. 6a), when penetrating the lens capsule (Fig. 6b) and in the lens (Fig. 6c). The penetration process from *corpus vitreum* into the lens through the capsule was associated with a slight injury to the lens capsule after penetration (Fig. 6c). At this early stage of infection a larger part of the metacercariae were found in *corpus vitreum* compared to the lens.

**Table 3:** Number of *Diplostomum pseudospathaceum* diplostomules/metacercariae found in histological sections of skin (head section), CNS (medulla, medulla oblongata, brain) and eyes (*corpus vitreum* and lens) of rainbow trout exposed to cercariae. Neighbour sections of the same parasite are disregarded, so the numbers represent individual parasites. Only data shown for 2–7 hpi because no parasites were recorded at 1 hpi in the sections. *: The 288 sections comprised skin, other tissues and CNS. **: The 455 sections comprised both lens and *corpus vitreum*

	Sections examined	No. of parasites detected
Skin	288*	4
CNS	288*	6
Other tissues	288*	6
Eye *corpus vitreum*	455	180
Eye lens	455	51

### Molecular detection of *D. pseudospathaceum* DNA in rainbow trout brain and eyes

*Determination of qPCR detection sensitivity and efficiency.* A ten-fold dilution series demonstrated 100% detection accuracy, with all eight replicates yielding positive amplification in dilutions from 100 to 10^− 4^. The detection rate decreased to 87.5% at the 10^− 5^ dilution and further dropped to 37.5% at the 10^− 6^ dilution. No detection was achieved at 10^− 7^ and 10^− 8^ dilutions (S2.1. Suppl File Fig. 1). Therefore, in the parasite detection assays, we used the mean Cq (31.77) value obtained from eight replicates from 10^− 4^ as threshold and any Cq value above was considered as negative (S2.1 Suppl File Table 1). Additionally, in the testing assays we also detected some amplification with fish DNA (Cq: 35 to 40) but all NTCs were negative. The standard curve obtained from fold-dilutions also demonstrated a strong linear relationship between DNA concentration and Cq values, indicating good analytical performance. Moreover, the assay revealed strong linearity (R² = 0.983) and an amplification efficiency of approximately 104% (S2. Suppl File S2.1 - Fig. 1).

Parasite DNA detection by quantitative realtime PCR was successful in brain (Table 4) and eyes (Table 5). The relative occurrence of the parasites was indicted by the Cq value. The parasite DNA was first found in lens and *corpus vitreum* 1 hpi and it increased steadily until termination at 7 hpi. Parasite detection in brain tissue followed the same pattern.

Thus, parasite DNA was detected at all time points in both brain and eye samples. In the infected brain samples, DNA amplification occurred in all samples and Cq values were below the established threshold. Initially (1hpi), the mean Cq value was 24.45. This value decreased during the course of infection reaching a mean Cq value of 21.14 at the final time point (7hpi) (Table 4).

**Table 4:** Molecular detection of *D. pseudospathaceum* DNA in fish brain. Infection level expressed by Cq values (low refers to high infection and high a low infection), hpi: Hours post infection

Brain			
Time point	Positive	Negative	Cq (Mean)
1 hpi	3	0	24.45
2 hpi	3	0	23.84
3 hpi	3	0	20.72
4 hpi	3	0	20.27
5 hpi	3	0	21.99
6 hpi	3	0	21.10
7 hpi	3	0	21.14

Similarly, in the infected eye samples, parasite DNA was detected at all time points, however 3 out of 70 samples were above threshold and 1 sample was not amplified. At 1hpi, the Cq values for both the *corpus vitreum* (CV) and lens were 21.81 and 29.02, respectively. At the later time points, Cq values decreased systematically over time, which indicated a higher parasite load (Table 5).

**Table 5:** Molecular detection of *D. pseudospathaceum* DNA in fish eyes. Infection level expressed as Cq values. hpi: Hours post infection

Eye						
	*Corpus vitreum*			Lens		
Time point	Positive	Negative	Cq (mean)	Positive	Negative	Cq (mean)
1 hpi	4	1	21.81	4	1	29.02
2 hpi	5	0	21.02	4	1	27.78
3 hpi	5	0	19.46	5	0	26.88
4 hpi	5	0	18.10	4	1	24.60
5 hpi	5	0	17.43	5	0	25.03
6 hpi	5	0	16.99	5	0	24.20
7 hpi	5	0	16.69	5	0	22.71

In the control samples for fish brain, the detected DNA levels were above the threshold, and they were considered negative (S2. Suppl. File 2). In the control samples for fisheye, the DNA was extracted by combination of both CV and lens, which resulted in higher DNA load of fish (S3. Suppl. File 3).

### Expression of immune relevant genes in spleen, liver, brain and eyes of rainbow trout infected by *D. spathaceum*

The best combination of two reference genes was β-actin and ELF-1α for liver and spleen samples, whereas β-actin and ARP were the best for eyes and brain (Software Normfinder).

### Spleen

**Fig. 7:**
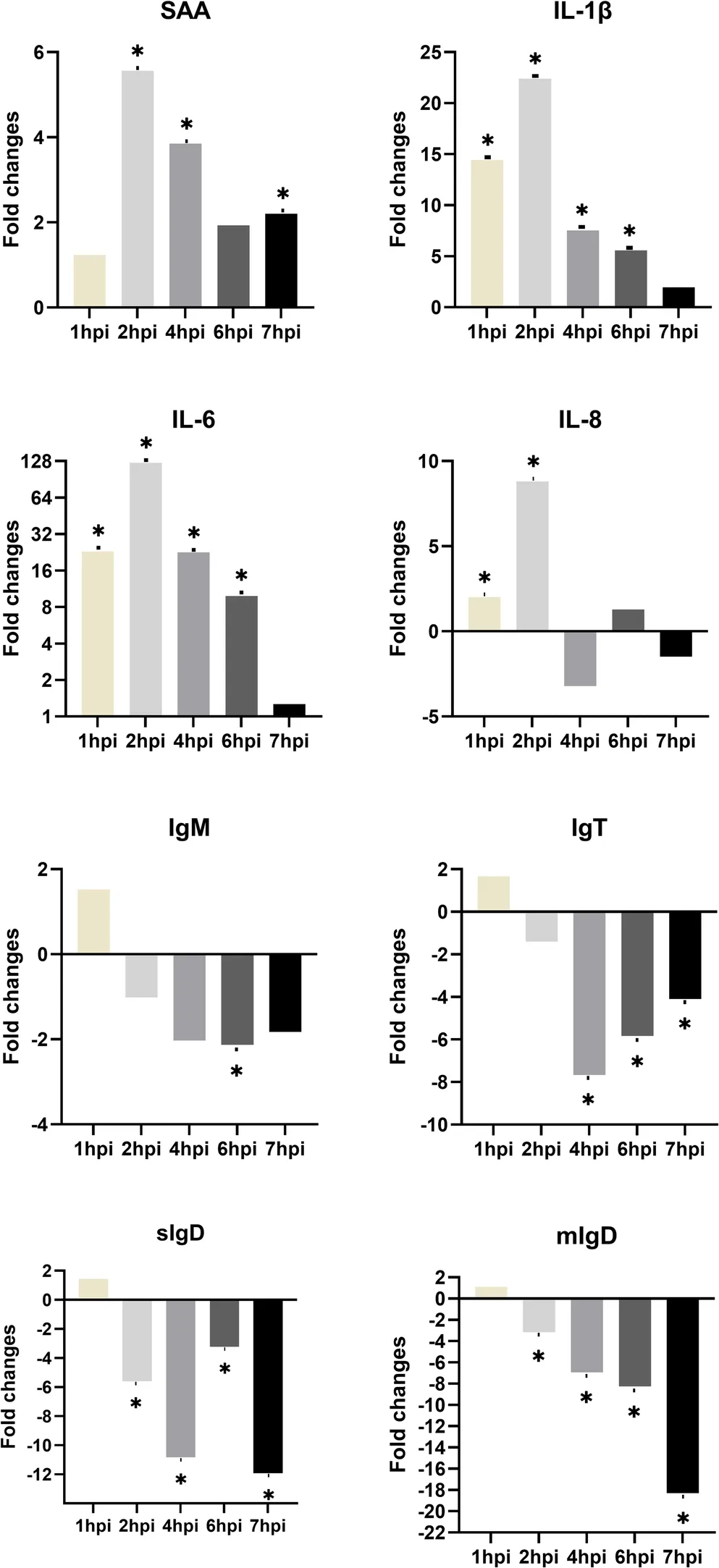
Expression of immune-relevant genes (selected) in spleen of rainbow trout exposed to *Diplostomum pseudospathaceum* cercariae over 7 h

During the infection and migration of *D. pseudospathaceum* from 1 to 7 hpi we recorded a swift and marked upregulation of genes encoding inflammatory markers IL-1β, IL-6, IL-8 and SAA, whereas genes encoding adaptive immune factors such as sIgD, mIgD, IgM, and IgT showed a significant down-regulation (Fig. 7).

### Liver

**Fig. 8:**
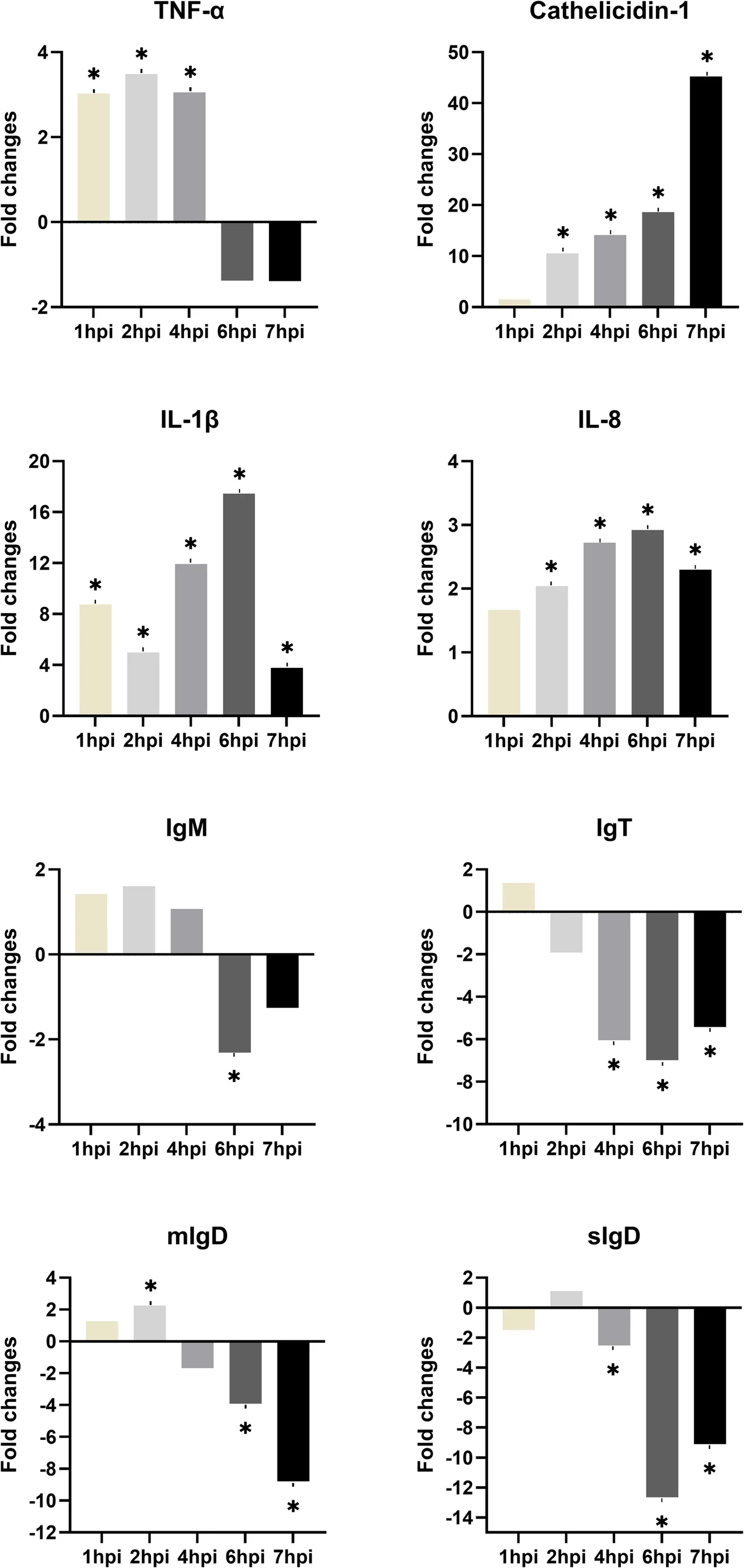
Expression of immune relevant genes (selected) in liver of rainbow trout exposed to *Diplostomum pseudospathaceum* cercariae over 7 h

During the infection genes encoding inflammatory markers TNF-α, IL-1β, IL-8 and the antimicrobial peptide cathelicidin1 were upregulated. In contrast, adaptive immune markers mIgD, sIgD, IgM and IgT were downregulated especially from 4 hpi (Fig. 8).

### Brain

**Fig. 9:**
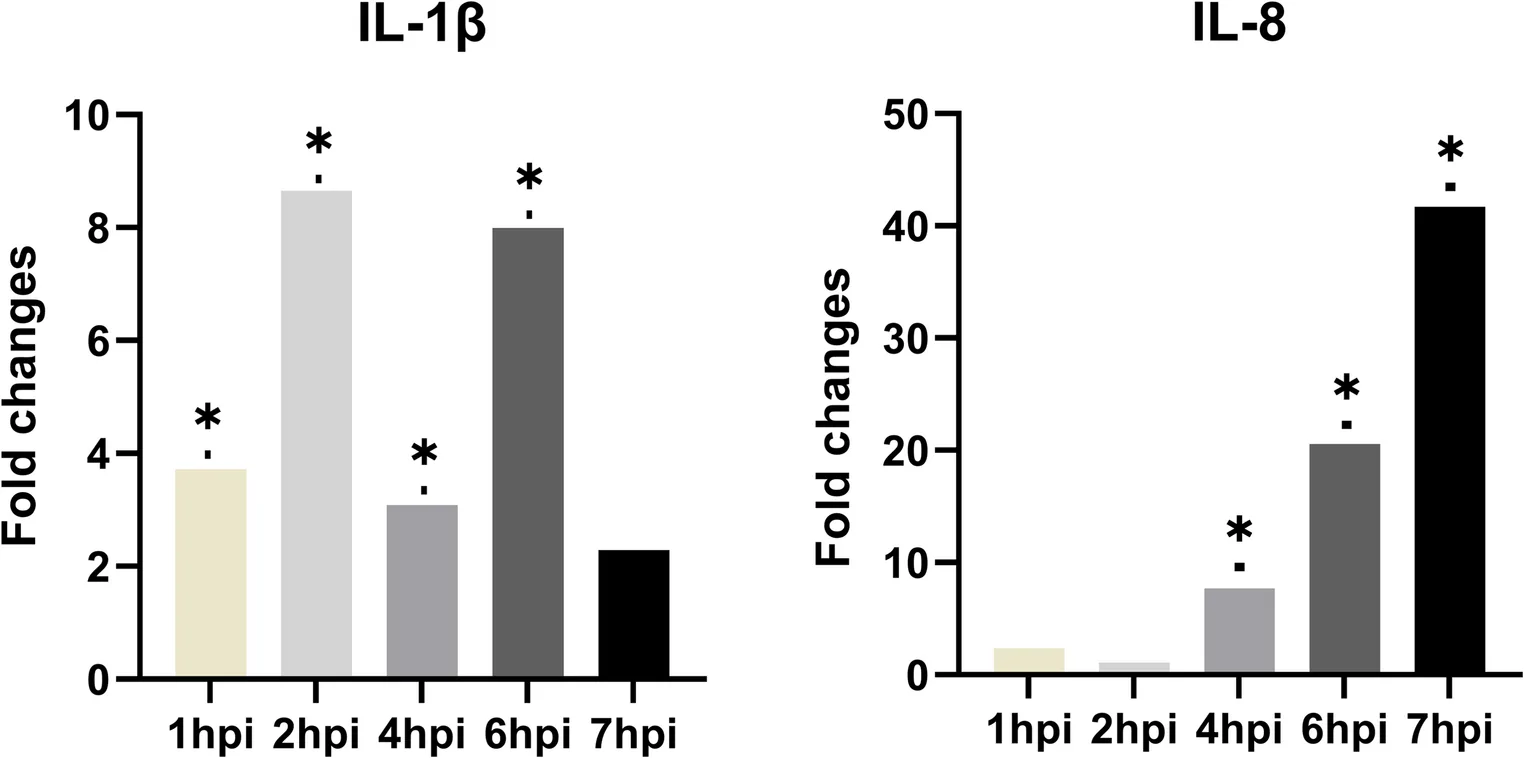
Expression of immune relevant genes (selected) in brain of rainbow trout exposed to *Diplostomum pseudospathaceum* cercariae over 7 h

During the infection fewer genes were regulated compared to the central immune organs (spleen and liver). Genes associated with a rapid innate inflammatory response (those encoding IL-1β, IL-6, IL-8 and the regulatory cytokine IL-10) were upregulated (Fig. 9) (Supplementary file S3.3).

### Eyes

It was noteworthy that merely few genes were regulated in the eyes despite a documented strong infection. In addition, a general trend for a non-significant down-regulation was seen for most genes (Supplementary file S3.4). Only *CD4* was upregulated at a late timepoint, whereas genes encoding TCR-β and mIgD were downregulated.

## Discussion

The present study has documented that the migration route of *D. pseudospathaceum* diplostomules in rainbow trout, following cercarial penetration of external surfaces, at least partly goes through CNS before the parasites reach the destination in the fish lens. We showed that some diplostomules followed a migration route from the subcutaneous skin layers via CNS (medulla, medulla oblongata, brain) before they reached the eye and entered *corpus vitreum* and the lens. The detection of migrating diplostomules in eye compartments (2 hpi) was more effective when using direct dissection of eyes compared to histological dissection (recorded 3 hpi). It may be due to a better fast over-all view of all eye-parts during dissection, whereas numerous histological sections (few micrometer thick) are more difficult to overview. The molecular detection method qPCR, histological observations and light microscopy of live larvae all showed early presence of the species in these host compartments.

The CNS pathway may offer an easier and less hostile environment for migrating parasites. This route of *D. pseudospathaceum* has not been reported previously, but a similar type of internal trematode migration has been described for *Trichobilharzia regenti* in mice (using 3D imaging) by Bulantova et al. (2016). It was later documented that the parasite migration in the spinal cord triggered immune reactions of the experimentally infected mice (Machacek et al. 2022). Cellular responses, including eosinophil infiltration, and a series of innate and adaptive immune factors were activated, whereas genes associated with optimal neurotransmission were downregulated.

Mice infected with *T. regenti* exhibited severe balance disturbances during parasite migration in brain tissue (Machacek et al. 2022), which may correspond to the equilibrium disturbances noted in *D. pseudospathaceum* infected rainbow trout documented in the present study. One major difference between the mouse/*T. regenti* study and the present fish/*D. pseudospathaceum* work is the timeline. The infection in the rodent spinal cord peaked at 7 days post-infection (dpi), whereas the rainbow trout infection in this study was at its highest level at 7hpi, where a large part of the diplostomules already had reached the ocular location. This suggests that the immune system in mice is activated over a longer time span, whereas the rainbow CNS represents a transient and not a lasting location. This may explain immunological differences noted between the two studies.

Migration routes of various species within the genus *Diplostomum* have been subject to debate (Ferguson 1943; Betterton 1974; Hoffman and Hoyme 1958; Erasmus 1959; Haas et al. 2007; Höglund 1991). The family diplostomidae comprises a wide range of species and ecotypes (Galazzo et al. 2002; Georgieva et al. 2012; Locke et al. 2009, 2015), which may have different host finding systems, invasion and protection mechanisms (Scharsack and Kalbe 2014). Accordingly, it can be speculated that different species exhibit varying behavioural patterns and follow various routes of migration to the eye in order to attain the metacercarial stage (Haas 1974; 1975; 2003). This would align with observation of differential pathogenicity of different diplostomids suggesting that diplostomules of different *Diplostomum* species show differential predilection for host organs and migration paths whereby some may be trapped before they reach the eye lens (Larsen et al. 2005). The present investigation used only one species, one strain and one individual clone of the parasite released from one snail. We can therefore not exclude that other individual clones of the same parasite species, but released from another snail host, may behave differently.

Based on our investigations we suggest that the CNS (medulla, medulla oblongata, brain, nervus opticus) may serve as an easy pathway to the eyes of the fish, whereby the most successful diplostomules along this route escapes the host immune system. This does not exclude that other less successful diplostomules become trapped in other and more exposed host locations. Thus, various authors have suggested that *D. spathaceum* diplostomules follow the vascular system when searching the eye location (Ferguson 1943; Betterton 1974; Höglund 1991).

The study lends support to the widely held hypothesis that the lens location in fish is an immune-priviledged site (Whyte et al. 1991; Fuad et al. 2024). Thus, infiltration into the delicate lens layers with cellular immune factors (granulocytes, macrophages, lymphocytes) and humoral elements (antibodies, complement and SAA) would disturb the anatomical integrity of the lens structure and thereby jeopardize host vision. In contrast, when the diplostomule occurs in other organs and tissues it is directly exposed to the immunological armament of the host (Bortz et al. 1984; Speed and Pauley 1985; Stables and Chappell 1986; Höglund and Thuvander 1990; Karami et al. 2022; Fuad et al. 2024). The parasite would therefore benefit from a migration route depleted of these major immune attacks.

It may then be discussed if the fish CNS is free from immune reactions. It was recently visualized by immune-histochemistry that lymphocytes positive for IgT and IgD occur in parts of the rainbow trout brain (Vicente-Gil et al. 2025), which would question the immune-priviledged status of the brain. However, the present investigation using quantitative realtime PCR of immune relevant genes indicated a much lower immune-reactivity in rainbow CNS (despite infection with diplostomules) compared to liver and spleen. We indicated with the qPCR work on brain tissue exposed to infection that diplostomules induce a slight inflammatory reaction involving IL-1β and IL-8 but leave other immune relevant genes (including a range of adaptive immune molecules) unregulated. In eyes the response was even further reduced as all immune genes remained un-regulated and we even saw a trend (although non-significant) for a general down-regulation.

We expect that not all cercariae find the optimal route, and our qPCR data on spleen and liver indicate that other diplostomules may be trapped on their way. Thus, in these organs a marked upregulation of inflammatory cytokines IL-1β, IL-8, IL-6 and SAA with further involvement of lysosome was noted. So, despite the selection of immunopriviledged locations by the diplostomules it is likely that some reaction may be traced in the fish host, which was also suggested by Fuad et al. (2024).

Immune-evasion of parasites can be the search for immunopriviledged host sites, but the invading diplostomule may have other tools to survive. Even in liver and spleen we noted that genes encoding adaptive immune factors such as IgM, IgD and IgT to a large extent were unregulated or down-regulated. This suggests that other well developed immune evasion mechanisms (such as immune-depressing molecules) are being applied as a survival strategy of *D. pseudospathaceum.*

## Conclusion

The present study demonstrated that cercariae of the eyefluke *Diplostomum pseudospathaceum* readily penetrated the skin of rainbow trout and migrated as diplostomules to the eye of the host within two hours. We present evidence that a part of the parasites finds an easy and less dangerous migration route by invading the CNS (moving along the *medulla*,* medulla oblongata*, brain) before reaching the eye, and finally move along *nervus opticus* before entering the vitreous humor and finally the lens. We do not exclude that some diplostomules do not find and apply this immunopriviledged migration route and remain in other host locations. This would activate the host immune reactions, and such a scenario was indeed documented in spleen and liver in the present study. The immune reactions in CNS and eye compartments (*corpus vitreum* and lens) were significantly lower.

## Supplementary Information

Below is the link to the electronic supplementary material.

Supplementary Material 1

Supplementary Material 2

Supplementary Material 3

Supplementary Material 4

Supplementary Material 5

Supplementary Material 6

Supplementary Material 7

Supplementary Material 8

## Data Availability

All data generated in this study is available in the submitted manuscript file and the supplementary files.
