# Supplementary material for: CNS migration of Diplostomum pseudospathaceum in rainbow trout: Neuro-immunological interactions
Source: Parasitol Res. 2026 Aug 5;125(1):140. doi: 10.1007/s00436-026-08741-y (PMC13736442; doi:10.1007/s00436-026-08741-y)
Supplement: Supplementary file 1 [file 436_2026_8741_MOESM1_ESM.docx]

**Supplementary file S1.** Table with primers and probes used for qPCR assays. All nucleotides are from 5’ end (labeled with FAM) to 3’ end (labeled with BHQ1). All the qPCR assays were optimized to have annealing temperature of 60°C and having efficiencies of 100% ± 5%. ^R^ indicates reference genes (housekeepers). ^MS^ indicates the qPCR assay targets both membrane bound and secreted forms. ^1^ The α chain of IL-12 is common to the two isoforms of IL-12.

| **Gene**  **GenBank acc.no.** | **Length**  **Bp** | **Primers**  **5’end to 3’end** | **Probes**  **5’end to 3’end** | **References** |
| --- | --- | --- | --- | --- |
| ^R^ ARP  AY505012 | 106 | Fwd: GAAAATCATCCAATTGCTGGATG  Rev: CTTCCCACGCAAGGACAGA | CTATCCCAAATGTTTCATTGTCGGCGC | [1] |
| ^R^ β-actin  AB196465 | 241 | Fwd: ACATCAAGGAGAAGCTGTGCTAC  Rev: TACGGATGTCCACGTCACAC | CCTCTCTGGAGAAGAGCTACGAGCTG | [2]  Probe [3] |
| ^R^ ELF-1α  [AF498320](http://www.ncbi.nlm.nih.gov/entrez/viewer.fcgi?db=nucleotide&val=20269865) | 63 | Fwd: ACCCTCCTCTTGGTCGTTTC  Rev: TGATGACACCAACAGCAACA | GCTGTGCGTGACATGAGGCA | [4] |
| C3.3 & C3.4  AF271080 / U61753 | 85 | Fwd: ATTGGCCTGTCCAAAACACA  Rev: AGCTTCAGATCAAGGAAGAAGTTC | TGGAATCTGTGTGTCTGAACCCC | [5] |
| Cathelicidin 1A  AY382478 | 189 | Fwd: TCTCTCGTCCTGGGGTT  Rev: GTTGTAGCGTGCTGATCTATG | TAATTGGTCGTCCTGGGGGTGG | [3] |
| CD4  AY973028 | 89 | Fwd: CATTAGCCTGGGTGGTCAAT  Rev: CCCTTTCTTTGACAGGGAGA | CAGAAGAGAGAGCTGGATGTCTCCG | [6] |
| CD8  AF178054 | 74 | Fwd: ACACCAATGACCACAACCATAGAG  Rev: GGGTCCACCTTTCCCACTTT | ACCAGCTCTACAACTGCCAAGTCGTGC | [7] |
| IFN γ1 and IFN γ2  FJ184374 / FJ184375 | 68 | Fwd: AAGGGCTGTGATGTGTTTCTG  Rev: TGTACTGAGCGGCATTACTCC | TTGATGGGCTGGATGACTTTAGGA | [8] |
| IgDm  AY870262 | 304 | Fwd: CAGGAGGAAAGTTCGGCATCA  Rev: CCTCAAGGAGCTCTGGTTTGGA | CCACACCACACAGACTCTGGCCCTGAA | [9] |
| IgDs  JQ003979 | 304 | Fwd: TGGCACGCCAGGATTTGAC  Rev: TCAGAATTGAGTGAACGGACAGACA | CCACACCACACAGACTCTGGCCCTGAA | [9] |
| ^MS^ IgM  S63348 / AH014877 | 72 | Fwd: ACCCTCCTCTTGGTCGTTTC  Rev: TGATGACACCAACAGCAACA | TGATGACACCAACAGCAACA | [8] |
| ^MS^ IgT  AY870265 / AY870263 | 73 | Fwd: AGCACCAGGGTGAAACCA  Rev: GCGGTGGGTTCAGAGTCA | AGCAAGACGACCTCCAAAACAGAAC | [8] |
| IL-1β  AJ223954 | 91 | Fwd: ACATTGCCAACCTCATCATCG  Rev: TTGAGCAGGTCCTTGTCCTTG | CATGGAGAGGTTAAAGGGTGGC | [8] |
| IL-2a  FJ571513 | 110 | Fwd: ATGCAACACCACATCAGCAT  Rev: TGCCACGGCCCTACAAAAGA  RE  TGCCACGGCCCTACAAAAGA | TGCCACGGCCCTACAAAAGA | [3] |
| IL-4/13a  AB574337 | 138 | Fwd: ATCCTTCTCCTCTCTGTTGC  Rev: GAGTGTGTGTGTATTGTCCTG | CGCACCGGCAGCATAGAAGT | [10] |
| IL-6a  DQ866150 | 91 | Fwd: ACTCCCCTCTGTCACACACC  Rev: *GGCAGACAGGTCCTCCACTA* | CCACTGTGCTGATAGGGCTGG | [11] |
| IL-8 isoforms a, b , c, d & e  AY160982 to AY160986 | 69 | Fwd: AGAATGTCAGCCAGCCTTGT  Rev: TCTCAGACTCATCCCCTCAGT | TTGTGCTCCTGGCCCTCCTGA | [11] |
| IL-10a  [AB118099](http://www.ncbi.nlm.nih.gov/entrez/viewer.fcgi?db=nucleotide&val=47678892) | 70 | Fwd: CGACTTTAAATCTCCCATCGAC  Rev: GCATTGGACGATCTCTTTCTTC | CATCGGAAACATCTTCCACGAGCT | [8] |
| ^1^ IL-12 α chain  HE798148 | 84 | Fwd: CAACGGAACACCACATTCAG  Rev: AGCCTGTAGTGAGGCAGCAT | TGCGTGTCTGAGGAACATCCG | [12] |
| IL-17A/F2a  AJ580842 | 158 | Fwd: TCAAAAGCAACGTGTCGAAG  Rev: TCCCTCTGATTCCTCTGTGG | TATGCTGCTGGGCCTGACCA | [12] |
| IL-17c1  CAW30792 | 138 | Fwd: CTGGCGGTACAGCATCGATA  Rev: GAGTTATATCCATAATCTTCGTATTCGGC | CGTGATGTCCGTGCCCTTTGACGATG | [10] |
| IL-17c2  CAW30793 | 134 | Fwd: CTGGCGGTACAGCATCGATA  Rev: CAGAGTTATATGCATGATGTTGGGC | CGTGGTGTCCAGGCCCTTTAATGATG | [10] |
| IL-22  AM748537 | 64 | Fwd: ATGACCACCACCACAGCATT  Rev: ATTCCTTTCCCCTCCTCCAT | CTTTCCGCAAGAAGTTGTCCGAG | [7] |
| Lysozyme  X59491 | 188 | Fwd: GAAACAGCCTGCCCAACT  Rev: GTCCAACACCACACGCTT | ATACCCAGGCCACCAACCGCAACAC | [13] |
| SAA  AM422446 | 79 | Fwd: GGGAGATGATTCAGGGTTCCA  Rev: TTACGTCCCCAGTGGTTAGC | TCGAGGACACGAGGACTCAGCA | [14] |
| TCR-β  AF329700 | 73 | Fwd: TCACCAGCAGACTGAGAGTCC  Rev: AAGCTGACAATGCAGGTGAATC | CCAATGAATGGCACAAACCAGAGAA | [8] |
| TNF-α1 & TNF-α2  AJ277604 / AJ401377 | 75 | Fwd: GGGGACAAACTGTGGACTGA  Rev: GAAGTTCTTGCCCTGCTCTG | GACCAATCGACTGACCGACGTGGA | [11] |

[1] Purcell MK, Kurath G, Garver KA, Herwig RP, Winton JR. Quantitative expression profiling of immune response genes in rainbow trout following infectious haematopoietic necrosis virus (IHNV) infection or DNA vaccination. *Fish Shellfish Immunol*. (2004) 17:447-62. [doi: 10.1016/j.fsi.2004.04.017](https://doi.org/10.1016/j.fsi.2004.04.017)

[2] Sugiura SH, Kelsey K, Ferraris RP. Molecular and conventional responses of large rainbow trout to dietary phosphorus restriction. *J Comp Physiol B*. (2007) 177:461-72. [doi: 10.1007/s00360-007-0144-9](https://doi.org/10.1007/s00360-007-0144-9)

[3] Zuo S, Karami AM, Ødegård J, Mathiessen H, Marana MH, Jaafar RM, et al. Immune gene expression and genome-wide association analysis in rainbow trout with different resistance to *Yersinia ruckeri* infection. *Fish Shellfish Immuno*l. (2020) 106:441-50. [doi: 10.1016/j.fsi.2020.07.023](https://doi.org/10.1016/j.fsi.2020.07.023)

[4] Ingerslev HC, Pettersen EF, Jakobsen RA, Petersen CB, Wergeland HI. Expression profiling and validation of reference gene candidates in immune relevant tissues and cells from Atlantic salmon (*Salmo salar* L.). *Mol Immunol.* (2006) 43:1194-201. [doi: 10.1016/j.molimm.2005.07.009](https://doi.org/10.1016/j.molimm.2005.07.009)

[5] Raida MK, Buchmann K. Innate immune response in rainbow trout (*Oncorhynchus mykiss*) against primary and secondary infections with *Yersinia ruckeri* O1. *Dev Comp Immunol*. (2009) 33:35-45. [doi: 10.1016/j.dci.2008.07.001](https://doi.org/10.1016/j.dci.2008.07.001)

[6] Raida MK, Buchmann K. Development of adaptive immunity in rainbow trout, *Oncorhynchus mykiss* (Walbaum) surviving an infection with *Yersinia ruckeri*. *Fish Shellfish Immunol*. (2008) 25:533-41. [doi: 10.1016/j.fsi.2008.07.008](https://doi.org/10.1016/j.fsi.2008.07.008)

[7] Olsen MM, Kania PW, Heinecke RD, Skjoedt K, Rasmussen KJ, Buchmann K. Cellular and humoral factors involved in the response of rainbow trout gills to *Ichthyophthirius multifiliis* infections: molecular and immunohistochemical studies. *Fish Shellfish Immunol*. (2011) 30:859-69. [doi: 10.1016/j.fsi.2011.01.010](https://doi.org/10.1016/j.fsi.2011.01.010)

[8] Raida MK, Buchmann K. Temperature-dependent expression of immune-relevant genes in rainbow trout following *Yersinia ruckeri* vaccination. Dis. Aquat Organ. (2007) 77:41-52. [doi: 10.3354/dao01808](https://doi.org/10.3354/dao01808)

[9] Skov J, Chettri JK, Jaafar RM, Kania PW, Dalsgaard I, Buchmann K. Effects of soluble immunostimulants on mucosal immune responses in rainbow trout immersion-vaccinated against *Yersinia ruckeri*. *Aquaculture*. (2018) 492:237-46. [doi: 10.1016/j.aquaculture.2018.04.011](https://doi.org/10.1016/j.aquaculture.2018.04.011)

[10] Chettri JK, Kuhn JA, Jaafar RM, Kania PW, Møller OS, Buchmann K. Epidermal response of rainbow trout to *Ichthyobodo necator*: immunohistochemical and gene expression studies indicate a T h1‐/T h2‐like switch. *J Fish Dis*. (2014) 37:771-83. [doi: 10.1111/jfd.12169](https://doi.org/10.1111/jfd.12169)

[11] Raida MK, Buchmann K. Bath vaccination of rainbow trout (*Oncorhynchus mykiss* Walbaum) against *Yersinia ruckeri*: effects of temperature on protection and gene expression. *Vaccine*. 2008 26:1050-62. [doi: 10.1016/j.vaccine.2007.12.029](https://doi.org/10.1016/j.vaccine.2007.12.029)

[12] Jaafar RM, Chettri JK, Dalsgaard I, Al-Jubury A, Kania PW, Skov J, Buchmann K. Effects of adjuvant Montanide™ ISA 763 A VG in rainbow trout injection vaccinated against *Yersinia ruckeri*. *Fish Shellfish Immunol*. (2015) 47:797-806. [doi:10.1016/j.fsi.2015.10.023](https://doi.org/10.1016/j.fsi.2015.10.023)

[13] Chettri JK, Raida MK, Kania PW, Buchmann K. Differential immune response of rainbow trout (*Oncorhynchus mykiss*) at early developmental stages (larvae and fry) against the bacterial pathogen *Yersinia ruckeri*. *Dev Comp Immunol*. (2012) 36:463-74. [doi: 10.1016/j.dci.2011.08.014](https://doi.org/10.1016/j.dci.2011.08.014)

[14] Skov J, Kania PW, Holten-Andersen L, Fouz B, Buchmann K. Immunomodulatory effects of dietary β-1, 3-glucan from *Euglena gracilis* in rainbow trout (*Oncorhynchus mykiss*) immersion vaccinated against *Yersinia ruckeri*. Fish Shellfish Immunol. 2012 33:111-20. [doi: 10.1016/j.fsi.2012.04.009](https://doi.org/10.1016/j.fsi.2012.04.009)
