# Supplementary material for: CNS migration of Diplostomum pseudospathaceum in rainbow trout: Neuro-immunological interactions
Source: Parasitol Res. 2026 Aug 5;125(1):140. doi: 10.1007/s00436-026-08741-y (PMC13736442; doi:10.1007/s00436-026-08741-y)
Supplement: Supplementary file 2 [file 436_2026_8741_MOESM2_ESM.docx]

**Suppl File 1, A**

**Figure 1.** Standard curve of ten-fold serial dilution of *D. pseudospathaceum.* The assay revealed strong linearity (R² = 0.983) and an amplification efficiency of approximately 104%.

**Suppl File 1, B**

**Table 1.** Cq values obtained from tenfold dilutions of *D. pseudospathaceum* in qPCR assay.

|  | **10^0^** | **10^-1^** | **10^-2^** | **10^-3^** | **10^-4^** | **10^-5^** | **10^-6^** | **10^-7^** | **10^-8^** | **Fish DNA** | **NTC** |
| --- | --- | --- | --- | --- | --- | --- | --- | --- | --- | --- | --- |
| **Replicate 1** | 17,47 | 21,85 | 25,55 | 28,37 | 31,77 | 36,12 | No Cq | No Cq | No Cq | No Cq | No Cq |
| **Replicate 2** | 17,92 | 21,69 | 24,77 | 28,41 | 31,66 | No Cq | 35,63 | No Cq | No Cq | 35,52 | No Cq |
| **Replicate 3** | 17,78 | 21,74 | 25,35 | 28,79 | 31,86 | 34,04 | No Cq | No Cq | No Cq | No Cq | No Cq |
| **Replicate 4** | 18,42 | 22,46 | 25,38 | 28,22 | 31,92 | 35,96 | No Cq | No Cq | No Cq | 38,98 | No Cq |
| **Replicate 5** | 18,32 | 21,71 | 25,37 | 28,55 | 31,55 | 34,13 | No Cq | No Cq | No Cq | 40 | No Cq |
| **Replicate 6** | 18,26 | 22,01 | 25,09 | 28,48 | 31,95 | 34,2 | No Cq | No Cq | No Cq | 39,79 | No Cq |
| **Replicate 7** | 18 | 21,7 | 24,88 | 28,2 | 31,44 | 34,08 | 35,52 | No Cq | No Cq | No Cq | No Cq |
| **Replicate 8** | 17,62 | 21,95 | 24,69 | 28,79 | 32 | 37,04 | 36,6 | No Cq | No Cq | No Cq | No Cq |
| **MEAN** | 17,97 | 21,89 | 25,14 | 28,48 | 31,77 | 35,08 | 35,92 | NA | NA | 38,57 | NA |
